# Simultaneous Determination of Schisandrin and Promethazine with Its Metabolite in Rat Plasma by HPLC-MS/MS and Its Application to a Pharmacokinetic Study

**DOI:** 10.1155/2019/3497045

**Published:** 2019-12-09

**Authors:** Sijia Gao, Xuelin Zhou, Liwei Lang, Honghong Liu, Jianyu Li, Haotian Li, Shizhang Wei, Dan Wang, Zhuo Xu, Huadan Cai, Yanling Zhao, Wenjun Zou

**Affiliations:** ^1^College of Pharmacy, Chengdu University of Traditional Chinese Medicine, Chengdu 611137, China; ^2^Department of Pharmacy, The Fifth Medical Center of PLA General Hospital, Beijing 100039, China; ^3^Department of Pharmacology, School of Basic Medical Sciences, Capital Medical University, Beijing 100069, China; ^4^The Center of Clinical Research, The Fifth Medical Center of PLA General Hospital, Beijing 100039, China; ^5^Department of Integrative Medical Center, The Fifth Medical Center of PLA General Hospital, Beijing 100039, China

## Abstract

This study aimed to develop a selective, simple, and sensitive HPLC-MS/MS method for the simultaneous determination of schisandrin and promethazine (PMZ) with its metabolite in rat plasma, which was further used for a pharmacokinetic herb-drug interaction study. HPLC-MS/MS analyses were performed on an Agilent Technologies 1290 LC and a 6410 triple quadrupole mass spectrometer. The following parameters, the lower limit of quantification (LLOQ), calibration curve, accuracy, precision, stability, matrix effect, and recovery, were validated. The linear range of the developed method for PMZ, its metabolite promethazine sulfoxide (PMZSO), and schisandrin in rat plasma was 0.5–200 ng/mL (*R*^2^ > 0.995), with an LLOQ of 0.5 ng/mL, which completely met the determination requirements of biosamples. The intra- and interday precision (RSD, %) was below 13.31% (below 16.67% for the LLOQ) in various plasma, whose accuracy (bias, %) was from −8.52% to 11.40%, which were both within an acceptable range. This method was successfully applied to a pharmacokinetic herb-drug interaction study after oral administration of PMZ with or without *S. chinensis* water extract. The results demonstrated that coadministration with the *S. chinensis* water extract might affect the pharmacokinetic behaviors of PMZ. In turn, when taken together with PMZ, the pharmacokinetic parameters of schisandrin, the main active component of *S. chinensis*, were also affected. The method established in the current study was selective, simple, sensitive, and widely available with good linearity, high accuracy and precision, and a stable sample preparation process. Moreover, this analytical method provides a significant approach for the investigation of herb-drug interaction between *S. chinensis* and PMZ. The potential pharmacokinetic herb-drug interaction of PMZ- and schisandrin-containing preparations should be noted.

## 1. Introduction

Vertigo, a sense of rotation or movement of the head and body, is a common and complex symptom that occurs in response to various factors [1, 2]. During the onset of vertigo, patients may feel the surrounding objects or themselves moving or rotating, and some patients may even have symptoms such as lightheadedness and dizziness, which seriously affect the life quality of patients [3]. Vertigo involves multiple medical disciplines, such as neurology, ophthalmology, and cardiovascular, and has become one of the key issues in clinical practice [4].

Benzodiazepines, antihistamines, anticholinergics, and monoaminergic agents are commonly used as the vertigo-inhibiting medications [5]. Promethazine (PMZ), one of the most frequently used phenothiazine derivatives, is primarily a histamine (H1) receptor antagonist, as well as a direct antagonist in muscarinic (M1) and dopamine (D2) receptors [6]; PMZ is considered the preferred option for vertigo [7]. It can contribute to the amelioration of nausea, vomit, and to some extent vertigo itself [8]. After oral administration, PMZ is metabolized in the liver and biotransformed to two major metabolites, including promethazine sulfoxide (PMZSO) and monodesmethyl promethazine sulfoxide, and a secondary metabolite, monodesmethyl promethazine [9], and finally excreted in the urine. CYP2D6 is closely associated with the hepatic metabolism of PMZ [10]. *Schisandra chinensis* is the dry fruits of *Schisandra chinensis* (Turcz.) Baill. (*S. chinensis*), also recognized as “Wu Wei Zi” in China [11]. It has become one of the most widely used traditional herbal medicines for thousands of years in China and other Asian countries. *S. chinensis*, with a variety of pharmacological effects, exhibited inhibitory or inductive effects on different hepatic cytochrome P450 enzymes (CYP450s) and may cause herb-drug interaction mediated by CYP450s [12]. In clinical practice, *S. chinensis* has a wide range of indications including vertigo, cough, asthma, insomnia, depression, hepatitis, jaundice, thirsty, shortness of breath, body weakness, palpitation, spontaneous sweating, and night sweating [13]. It can be used in combination with PMZ in the treatment of vertigo, cough, and allergic diseases. Schisandrin is a kind of high content lignan in *S. chinensis*, with the tranquillizing and sedative effects, which may be the potential basis for *S. chinensis* to change the liver drug enzyme activity, further affecting the pharmacokinetic characteristics of PMZ and its metabolites.

Several analytical methods have been reported for the pharmacokinetic determination in biological samples including high-performance liquid chromatography with ultraviolet (UV) detection [14], fluorescence detection (FD) [15], electrochemical detection (ECD) [16], photodiode array (PDA) detection [17], mass spectrometric (MS or MS/MS) detection [18, 19], and some chemiluminescence (CL) technique [20]. However, regarding HPLC-FD, complex sample pretreatment is required to decrease chromatographic interferences from the biological matrix. Due to the limited source of biological samples and high requirements for analyte concentration, a UV detection tool has been gradually replaced by LC-MS/MS in recent years [21]. ECD has been recognized as a valuable method with better sensitivity and selectivity compared to UV and FD methods for determination of electroactive compounds; however, the complex pretreatment procedure of biosamples and long analytical time limit its application. PDA detection has also been applied for pharmacokinetic determination, but with the disadvantages of low sensitivity and large sample volume requirements [22]. In the past decades, the use of LC-MS/MS for the determination of drugs and their metabolites has dramatically increased due to its unique advantages in improving selectivity, specificity, and sensitivity [23–25]. MS/MS detection could avoid the complex sample pretreatment procedure. Nevertheless, possible matrix effects in biological samples should be taken into consideration when using electrospray ionization (ESI) [26]. Then, internal standards are usually introduced for eliminating matrix effects and improving the accuracy when using the LC-ESI-MS/MS method for the pharmacokinetic determination of the target compound in biological samples. In this study, metronidazole was chosen as the internal standard for determination of PMZ and its main metabolite PMZSO and bifendate as the internal standard for schisandrin due to their high similarity to the analytes and both metronidazole and bifendate are absent in biological samples. In addition, the two internal standards selected are cheap and easy to obtain, stable in nature, and suitable for preservation.

To the best of our knowledge, no methods have been established for the simultaneous detection of schisandrin from *S. chinensis* and PMZ with its metabolite PMZSO due to complex biological matrices. The high-performance liquid chromatography-tandem mass spectrometry (HPLC-MS/MS) method with highly effective separation and good specificity and sensitivity, along with short analytical time and low sample consumption, may be a preferred option for simultaneous determination of multiple metabolites *in vivo*. Therefore, the aim of this study was to develop a selective, simple, and sensitive HPLC-MS/MS method for simultaneous determination of schisandrin, PMZ, and PMZSO in rat plasma and furthermore for pharmacokinetic herb-drug interaction evaluation. After optimization and validation, the established method was applied to analyze the plasma samples of rats after oral administration of PMZ and *S. chinensis*. Afterwards, the compounds were simultaneously determined in plasma samples, and their concentration-time curves were constructed separately. The results indicated that potential pharmacokinetic (PK) herb-drug interaction of PMZ- and schisandrin-containing preparations should be noted in clinics.

## 2. Materials and Methods

### 2.1. Chemicals and Reagents

**Figure 1:**
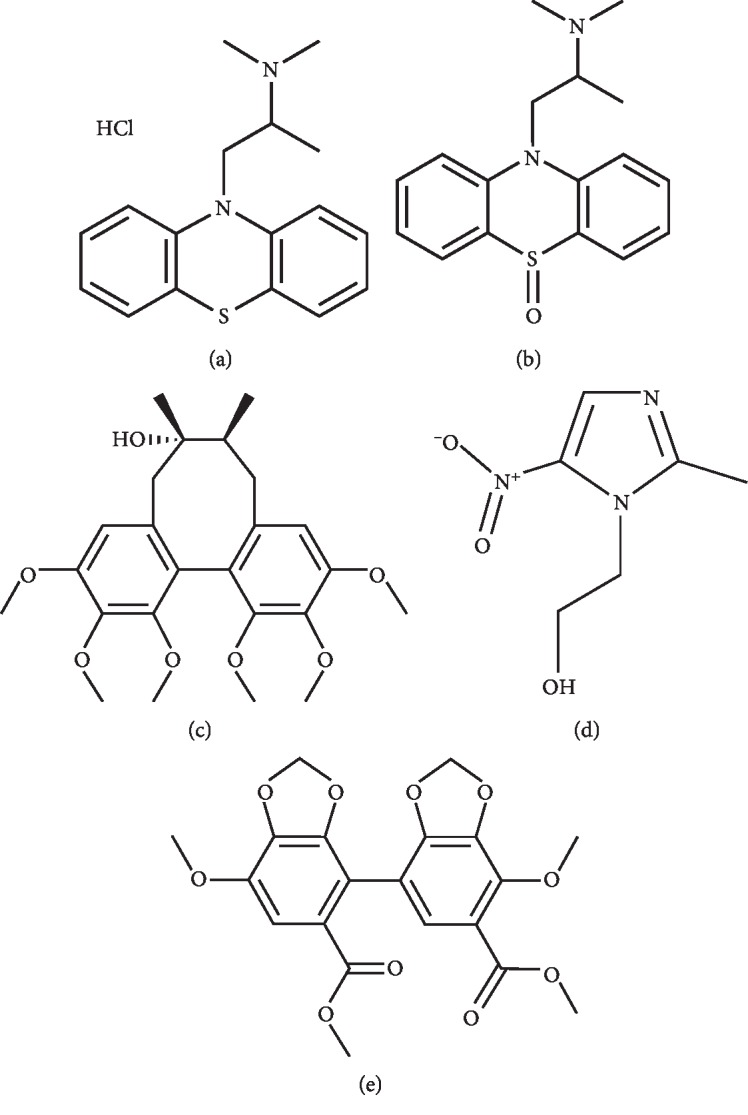
Chemical structure of (a) promethazine hydrochloride, (b) PMZSO, (c) schisandrin, (d) metronidazole, and (e) bifendate.

Promethazine hydrochloride (Batch No. RNB9368V, purity >98%) was purchased from Sigma-Aldrich Co. (St. Louis, MO, USA). Promethazine sulfoxide (Batch No. 1-JGC-73-2, purity >98%) was manufactured by Toronto Research Chemicals (Toronto, Canada). Metronidazole (Batch No. ALT600205, purity >98%) was provided by A Chemtek (Worcester, Ma, USA). Standards of schisandrin (Batch No. 110857-201211, purity >98%) and bifendate (Batch No. 100192-201504, purity >98%) were obtained from the National Institutes for Food and Drug Control (Beijing, China). The chemical structures of each reference substances are shown in Figure 1. Acetonitrile (ACN) and methanol (MeOH) used in this study were of LC-MS grade (Fisher Chemical Company, Geel, Belgium). Deionized water was purified by a Millipore water purification system (Millipore, Billerica, MA, USA). Formic acid was purchased from Sigma-Aldrich Co.

### 2.2. Herbal Extraction

*S. chinensis* (Batch No. 18011201) was supplied by Beijing Lvye Medicinal Materials Company (Beijing, China), which was strictly authenticated in accordance with the Chinese Pharmacopoeia (Edition 2015, Volume I). *S. chinensis* was soaked in water (1 : 10, *w/v*) for 30 min, that water was extracted for 2 h, and this process was repeated again. The water extract of *S. chinensis* was merged together, filtered, and evaporated until dry under negative pressure to prepare freeze-dried powder. The extraction yield was 43.15%. The freeze-dried powder was dissolved in deionized water, and the final concentration of the water extract was 0.56 g/mL.

### 2.3. Experimental Animals and Drug Administration

Male Sprague-Dawley rats (250–280 g) were supplied by the Animal Center of Beijing Charles River Co. (Beijing, China). Animals were housed with alternating 12 h light-dark cycles and had free access to rodent cubes and tap water. This study was performed in accordance with the Guiding Principles for the Care and Use of Laboratory Animals of China and approved by the Ethics Committee of Fifth Medical Center of PLA General Hospital (Ethics number SYXK 2017-0016).

For single treatment, rats were randomly divided into three groups: (1) PMZ (promethazine hydrochloride, 5.25 mg/kg), (2) WWZ (*S. chinensis* water extract, 2.8 g/kg), and (3) WWZ + PMZ (promethazine hydrochloride, 5.25 mg/kg, plus *S. chinensis* water extract, 2.8 g/kg). Blood samples were collected at 5, 10, 15, 30, and 45 min and 1, 2, 4, 6, 8, 12, and 24 h after drug administration.

For 3-week treatment, rats were divided into two groups: (1) WWZ (*S. chinensis* water extract, 2.8 g/kg, for 3 weeks) and (2) WWZ + PMZ (*S. chinensis* water extract, 2.8 g/kg, for 3 weeks and promethazine hydrochloride, 5.25 mg/kg, for the last administration). Blood samples were collected according to the above-described procedure.

### 2.4. Sample Preparation

All samples were thawed for at least 1 h to room temperature before formal preparation. Plasma samples (100 *μ*L) were spiked with 10 *μ*L of internal standard (IS) solution (consisting of 260.5 ng/mL metronidazole and 5740 ng/mL bifendate), and MeOH (300 *μ*L) was added for protein precipitation. The mixture was vortexed for 10 min by a multitube vortexer and centrifuged at 14800 rpm for 10 min at 4°C. The supernatant was collected and dried under a gentle flow of nitrogen gas at 34°C. The residue was reconstituted in 50% MeOH (100 *μ*L), vortexed for 10 min, and centrifuged at 14800 rpm for 10 min at 4°C. The supernatant (10 *μ*L) was injected into the LC-MS/MS system for further analysis.

### 2.5. LC-MS/MS Conditions

LC-MS/MS analyses were performed on an Agilent Technologies 1290 LC and an Agilent Technologies 6410 triple quadrupole mass spectrometer (Agilent Technologies, CA, USA). An electrospray ionization (ESI) source in the positive ionization mode was connected to the MassHunter Quantitative Analysis software (version B04.00).

The chromatographic separation was performed on a Zorbax Eclipse Plus C18 column (2.1 mm × 100 mm, 3.5 *μ*m, Agilent Technologies, CA, USA) at 25°C. The mobile phase consisted of H_2_O (containing 0.1% formic acid, A) and ACN (B) in the gradient elution program: 0 ⟶ 3 min, 17% B; 3 ⟶ 4.1 min, 17% ⟶ 40% B; 4.1 ⟶ 12 min, 40% B; and 12.1 ⟶ 13 min, 40% ⟶ 17% B. The flow rate was 0.35 mL/min, and the sample injection volume was 10 *μ*L.

The MS/MS analysis was performed under the multiple-reactions monitoring (MRM) mode. The MRM parameters of 3 target compounds and 2 internal standards are summarized in Table 1. The capillary voltage was set at 4000 V, and the gas temperature was at 300°C. The gas flow was 11 L/min, and the nebulizer was set to 15 psi.

### 2.6. Method Validation

Method validation was executed according to the USA FDA guidelines for bioanalytical method validation (Guidance for Industry: Bioanalytical Method Validation, 2013), and the following validation parameters were evaluated: specificity and selectivity, carryover effect, lower limit of quantification (LLOQ), calibration curve, accuracy, precision, stability, matrix effect, and recovery.

#### 2.6.1. Specificity and Selectivity

The specificity and selectivity were investigated by comparing LC-MS/MS MRM chromatograms of the blank plasma samples, the spiked plasma samples with the 3 analytes and internal standards, and the plasma samples obtained from PK studies.

#### 2.6.2. Carryover Effect

The carryover experiment was assessed by analyzing response of the blank plasma sample after the highest calibration standard (200 ng/mL). The acceptance criterion was carryover less than 20% for the LLOQ and 5% for the IS.

#### 2.6.3. Accuracy, Precision, and Linearity

Linearity was determined in the range of 0.5–200 ng/mL. The calibration curves were obtained from the peak area ratio of 3 analytes to internal standards against the nominal concentration of analytes. The correlation coefficient (*R*^2^) values of all calibration curves were required to be at least over 0.99. The accuracy (bias, %) was determined from the mean value of the observed concentration (*C*_obs_) and nominal concentration (*C*_nom_) by the following relationship: accuracy (bias, %) = ((*C*_obs_ − *C*_nom_)/*C*_nom_) × 100. The relative standard deviation (RSD, %) was calculated as follows: (standard deviation (SD)/*C*_obs_) × 100. Accuracy and precision were evaluated by determining the QC samples at three concentration levels and LLOQ samples in five replicates on the same day and on three consecutive days. Intraday and interday variations of the QC samples in the developed method were less than ±15% (±20% at the lower limit of detection) for all analytes.

#### 2.6.4. Stability

The stability of the analytes was assessed by analyzing three concentrations of the QC and LLOQ samples under the following storage conditions: freeze-thaw stability (stored at −80°C for 24 h and thawed to room temperature, for three cycles), short-term stability (kept at room temperature for 4 h), and postpreparative stability (kept in the autosampler at room temperature for 24 h).

#### 2.6.5. Recovery and Matrix Effect

The recovery and matrix effects of analytes in blood samples were assessed at three different concentrations (1.25, 12.5, and 150 ng/mL). The extraction recovery and matrix effect at three QC concentrations were measured. Recovery was calculated by comparing peak areas of three analytes (PMZ, PMZSO, and schisandrin) in the extracted QC samples with those in the postextracted blank plasma samples. The matrix effect was evaluated by comparing the peak area of the spiked samples after extraction with that of the neat solution's equivalent concentration.

### 2.7. Pharmacokinetic Analysis

The pharmacokinetics analysis was conducted by Drug and Statistics (DAS) 2.0 pharmacokinetic software program (Mathematical Pharmacology Professional Committee of China, Shanghai, China). The concentration-time curves were plotted. The time to reach the peak concentration (*T*_max_) and the maximum plasma concentration (*C*_max_) were obtained after oral administration. The results are shown as mean ± standard deviation (SD). One-way analysis of variance was used to calculate the differences. A *p* value less than 0.05 was considered a significant difference.

## 3. Results and Discussion

### 3.1. Optimization of the HPLC-MS/MS Conditions

**Figure 2:**
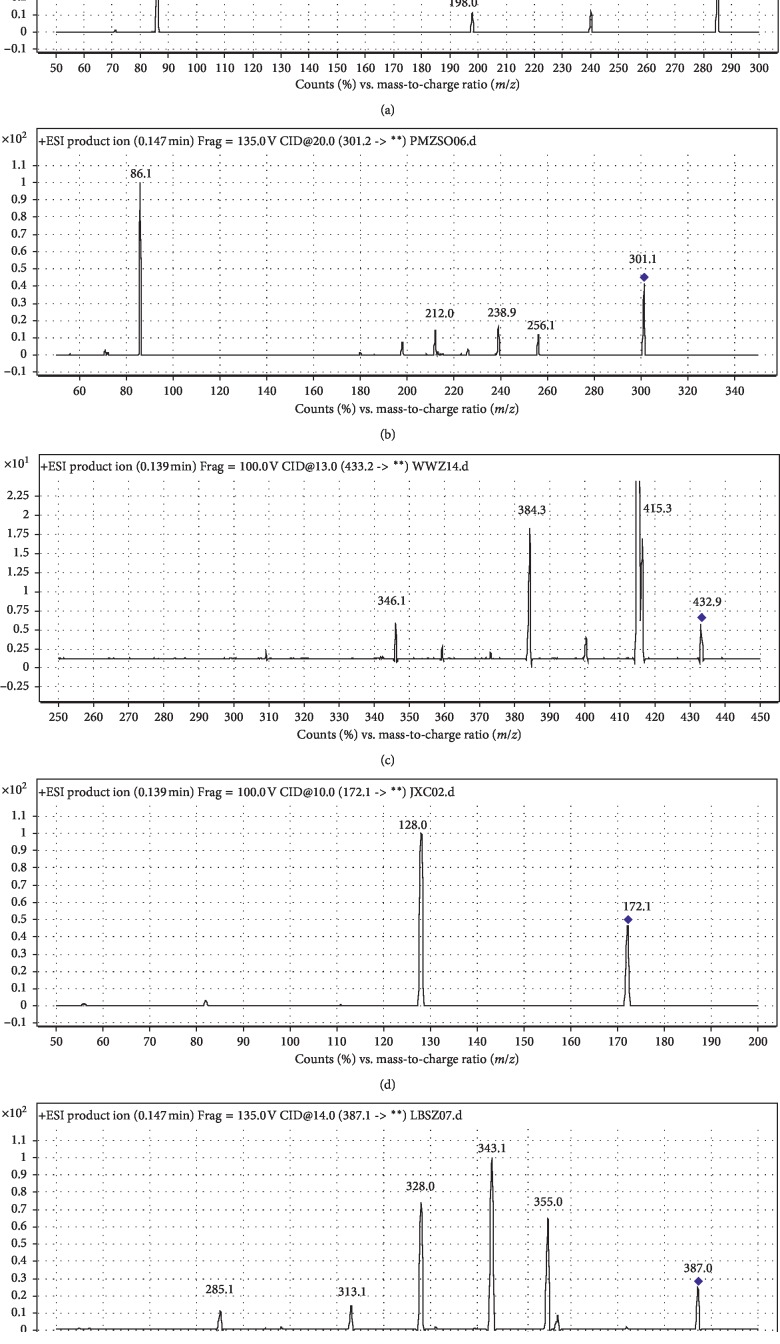
MS scans for (a) promethazine hydrochloride, (b) PMZSO, (c) schisandrin, (d) metronidazole, and (e) bifendate.

To evaluate the mass spectral fragmentation patterns of analytes and optimize the parameters, standard solutions of PMZ (100 ng/mL), PMZSO (100 ng/mL), and schisandrin (100 ng/mL) and two internal standards were analyzed by direct injection into the spectrometer, respectively. A full scan in the positive mode (*m/z* ranging from 50 to 500) was used to identify the analytes. The MS scans for PMZ, PMZSO, schisandrin, and two internal standards are shown in Figure 2.

The precursor ions and main product ions are shown in Table 1. The monitored precursor-product ions in the MRM mode provided high selectivity and sensitivity for quantification. Key parameters, such as fragmentor and collision energy, are also optimized in Table 1.

The mobile phase was optimized by comparing ACN/MeOH-water solvent systems. The results showed that the mobile phase comprising the ACN-water system exhibited higher response, lower background noise, and shorter analytical time when compared to MeOH-H_2_O composition. Regarding the reversed-phase chromatography, some acid, basic, or buffer solutions were usually added to maintain the pH value of the mobile phase for improving the peak shape and enhancing the resolution of targets. Therefore, 0.1% formic acid (v/v) was finally chosen to adjust the pH value so as to improve the peak symmetry, ionization effect, and sensitivity of the analytes.

### 3.2. Sample Preparation

Different solvents, such as MeOH, ACN, and MeOH-ACN (2 : 1, v/v), were used as protein precipitation reagents, and the extraction efficiency was compared. The results indicated that MeOH allowed for lower background noise and higher response with acceptable RSD (%) and bias (%) than ACN or MeOH-ACN (2 : 1, v/v). The liquid-liquid extraction method was also evaluated but finally not adopted because the polarities of the analytes varied widely, making it difficult to find an appropriate solvent to extract all analytes well. Thus, plasma samples were treated with MeOH as in the above-described procedure.

### 3.3. Method Validation

**Figure 3:**
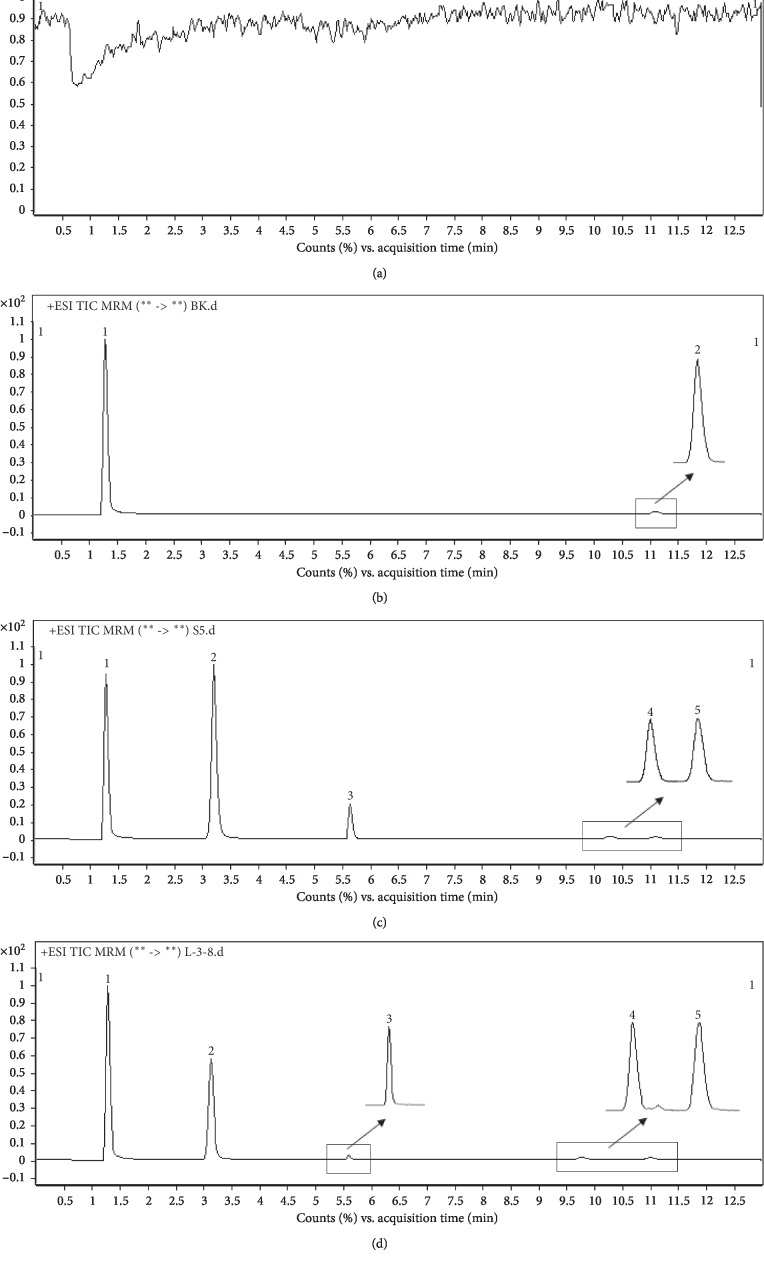
HPLC-MS/MS MRM chromatograms of (a) blank plasma, (b) spiked plasma with IS, (c) spiked blank plasma with PMZ, PMZSO, schisandrin, and IS samples, and (d) actual rat plasma samples collected at 1 h after oral administration. 1: metronidazole; 2: PMZSO; 3: PMZ; 4: schisandrin; 5: bifendate.

The results presented in Figure 3(a) show that no significant interferences from rat plasma were found at the retention times of PMZ, PMZSO, schisandrin, and IS, illustrating good selectivity of the established HPLC-MS/MS method.  Figure 3(b) shows blank sample spiked with internal standards, and Figure 3(c) shows blank plasma spiked with the standards of PMZ, PMZSO, and schisandrin. The chromatogram of the actual rat plasma sample collected at 1 h after oral administration is also presented in Figure 3(d).

The analysis of the blank plasma sample immediately after the highest calibration standard showed no interfering and residual peaks, revealing no carryover effect (Supplementary [Supplementary-material supplementary-material-1]).

Linear regression analysis of the calibration curves in blank plasma with five replicates over three different days indicated good linearity in the concentration range of 0.5–200 ng/mL. The correlation coefficients (*R*^2^) were greater than 0.995. The LLOQs for the three analytes were 0.5 ng/mL, as shown in Table 2.

The intra- and interday accuracies (bias, %) and precisions (RSD, %) of PMZ, PMZSO, and schisandrin were determined at 0.5, 1.25, 12.5, and 150 ng/mL, respectively. The results in Table 3 show that all the bias and RSD values were within ±15%, except for the LLOQ which was within ±20% which is still within an acceptable range. These findings indicated that the developed HPLC-MS/MS method was excellent for the simultaneous quantitative analysis of PMZ, PMZSO, and schisandrin in biological plasma samples.

Three concentrations of QC samples were determined for recovery and matrix effect evaluation. The concentrations of PMZ, PMZSO, and schisandrin were 1.25, 12.5, and 150 ng/mL and of metronidazole and bifendate were 5740 ng/mL and 260.5 ng/mL, respectively. The results in Table 4 indicate that the extraction recovery and matrix effect were 84.50–89.81% and 102.37–104.36% for PMZ, 96.70–100.44% and 121.56–122.80% for PMZSO, and 97.92–99.79% and 105.19–109.68% for schisandrin, respectively, which were acceptable according to the FDA's biological method validation guidelines. Therefore, the developed HPLC-MS/MS method was acceptable for the pharmacokinetic study of PMZ, PMZSO, and schisandrin in freely moving rats.

RSD values regarding stability of three analytes are shown in Table 5. Three concentrations of QC samples were evaluated for different storage conditions. 1.25, 12.5, and 150 ng/mL concentrations of PMZ, PMZSO, and schisandrin were applied for method validation. The results demonstrated that no significant degradation of analytes occurred in biological samples after storage in the autosampler for 24 h at room temperature, after short-term storage for 4 h at room temperature, and after three freeze-thaw cycles at −80°C and thawing at room temperature, respectively.

### 3.4. Pharmacokinetic Application

**Figure 4:**
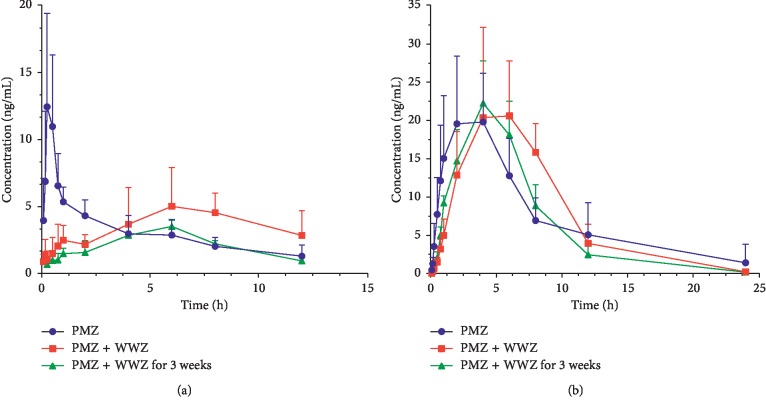
Mean plasma concentration-time curve of (a) PMZ and (b) PMZSO in rat plasma samples after oral administration of PMZ with or without *S. chinensis* (*n* = 6-7). Data were analyzed by one-way ANOVA. All data are presented as mean ± SD.

The developed and validated HPLC-MS/MS method was successfully applied for the determination of PMZ with its major metabolite and schisandrin in rat plasma after oral administration with or without coadministration of the *S. chinensis* water extract. The mean plasma concentration-time curves are illustrated in Figure 4, and the corresponding pharmacokinetic parameters are summarized in Table 6. It can be found that, after oral administration, PMZ was rapidly absorbed into plasma and reached its maximum plasma concentration (*C*_max_ = 14.35 ± 6.17 ng/mL) 0.36 h post-dose. PMZ was then eliminated from the body with a half-life of 5.92 h. When coadministered with the water extract of *S. chinensis*, the *C*_max_ of PMZ was 6.96 ± 2.12 ng/mL, significantly lower than that of the PMZ group. Compared with that of the *S. chinensis*-free group, the *T*_max_ of PMZ was significantly prolonged (*p* < 0.05). It is worth noting that coadministration of the *S. chinensis* water extract had no significant effects on AUC(0-*t*) and *t*_1/2_ of PMZ.

In clinics, most Chinese medicines are not used once, and they are often given for long time to achieve better efficacy, such as the commonly used *S. chinensis*. In order to further investigate the pharmacokinetic effects of *S. chinensis* on PMZ, the plasma concentration of PMZ after long-term administration of *S. chinensis* for three weeks was determined. As shown in Figure 4 and Table 6, after long-term use of the *S. chinensis* water extract, the AUC(0-*t*) of PMZ was decreased to 24.76 ± 3.45 ng h/mL. Compared with those of the single administration of PMZ, the *T*_max_ of PMZ was significantly increased to 5.67 ± 0.82 h and the maximum plasma concentration (*C*_max_) of PMZ was decreased to 3.59 ± 0.57 ng/mL. Meanwhile, as the main bioactive metabolite of PMZ, the concentration of PMZSO in rat plasma samples was simultaneously determined. The mean plasma concentration curve in Figure 4(b) indicates that, after oral administration of PMZ, the maximum plasma concentration of PMZSO reached 23.37 ng/mL at 3.43 h with a half-life (*t*_1/2_) of 3.35 h. Its pharmacokinetic parameters were not affected even after coadministration with the *S. chinensis* water extract. When the water extract of *S. chinensis* was given, the half-life was decreased to 2.55 h.

**Figure 5:**
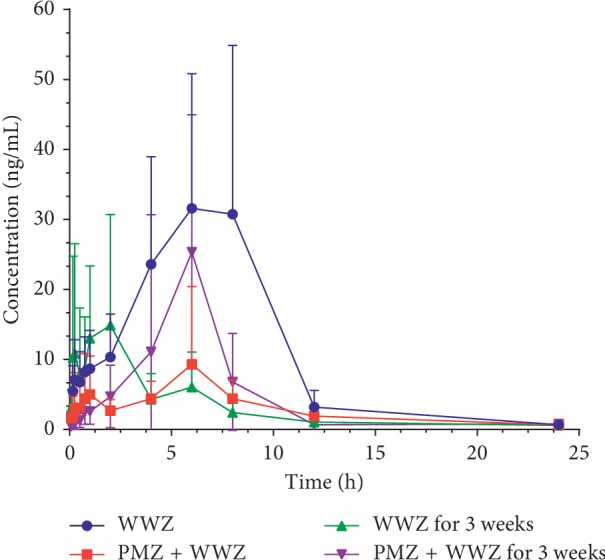
Mean plasma concentration-time curve of schisandrin after oral administration of the *S. chinensis* water extract once or for continuously three weeks, coadministered with or without PMZ (*n* = 6–7). Data were analyzed by one-way ANOVA. All data are presented as mean ± SD.

In turn, the concentration of schisandrin (one of the main active components of *S. chinensis*) in rat plasma samples was also determined. Interestingly, as shown in Table 7 and Figure 5, after coadministration with PMZ, the pharmacokinetic parameters of schisandrin were significantly decreased except for *t*_1/2_; that is, AUC(0-t) and *C*_max_ were decreased significantly compared with those of the PMZ-free group, and *t*_1/2_ was prolonged significantly. Considering that *S. chinensis* is a potential liver drug enzyme inhibitor or inducer, the pharmacokinetic effects of long-term (three weeks) administration of *S. chinensis* on its own active component were investigated. Through comparison, it was found that, after administration with the *S. chinensis* water extract for three weeks, the pharmacokinetic parameters of schisandrin changed significantly with the decreased AUC(0-t), *T*_max_, and *C*_max_. The results indicated that the water extract of *S. chinensis* may be a potential inhibitor of CYP450s and drug transporters, which can not only reduce the absorption of PMZ but also inhibit the absorption of schisandrin.

In clinics, both *S. chinensis* and PMZ are commonly used, and especially, *S. chinensis* as a common Chinese herbal tonic has been widely used in many traditional Chinese medicine prescriptions, making the combined use of two drugs more common. Drug-drug interaction (DDI) has become one of the most important factors affecting drug usage, which may cause serious side effects and further result in refusal of approval and even drug withdrawal from market [27]. Drugs may interact with some therapeutic agents by inhibiting or promoting drug-metabolizing enzymes; inhibition of drug-metabolizing enzymes can change their pharmacokinetic behaviors including leading to longer half-lives, higher exposures, and slower clearances of drugs and exert the potential toxicity [28]. Promoting drug-metabolizing enzymes may accelerate drug absorption and metabolism, further resulting in poor efficacy. Botanical dietary supplements or herbal medicine may affect the pharmacokinetic behaviors of drugs by inhibiting or promoting drug-metabolizing enzymes. The safety of herbs has drawn more attention, and herb-drug interactions (HDIs) are increasingly recognized as crucial clinical events [29, 30]. Pharmacokinetic interactions are one of the possible HDI factors. Exploration of pharmacokinetic interactions between drugs and herbs leads to a better understanding of adverse drug reactions and avoids their occurrence to a certain extent. Thus, in the current study, we established a rapid, sensitive, and selective method for the detection of schisandrin and PMZ with its sulfoxide to explore the potential pharmacokinetic interaction between *S. chinensis* and PMZ in order to further guide the clinical medication. The results showed that *S. chinensis*, especially after long-term use, significantly inhibited the absorption of PMZ, suggesting that *S. chinensis* may be a potential inhibitor against drug-metabolizing enzymes or drug transporters, affecting the absorption of drugs.

## 4. Conclusions

In this study, a rapid, sensitive, and selective HPLC-ESI-MS/MS method has been developed and validated for simultaneous determination of the major component of *S. chinensis* and PMZ with its metabolite in rat plasma. It was successfully applied to the preliminary pharmacokinetic study of herb-drug interaction between *S. chinensis* and PMZ. Besides, the potential pharmacokinetic herb-drug interaction of PMZ- and schisandrin-containing preparations should be noted.

## Figures and Tables

**Table 1:** MRM transitions, retention time, and conditions of analytes and internal standards.

Compound	Retention time (min)	Precursor ion	Product ion	Fragmentor (V)	Collision energy	Cell accelerator voltage
Promethazine	5.62	285.1	86.1	95	13	8
Promethazine sulfoxide	3.15	301.2	86.1	125	25	8
Metronidazole	1.47	172.1	128.1	100	10	5
Schisandrin	9.77	433.2	384.2	90	16	6
Bifendate	11.03	387.1	328	135	10	7

**Table 2:** Regression equations, linear ranges, and LLOQs of the three compounds.

Compound	Regression equation	*R* ^2^	Linear range (ng/mL)	Limit of quantification (ng/mL)
Promethazine	*Y* = 0.016464*X* + 0.001150	0.9983	0.5–200	0.5
Promethazine sulfoxide	*Y* = 0.041898*X* + 0.001187	0.9961	0.5–200	0.5
Schisandrin	*Y* = 0.054003*X* + 0.001437	0.9986	0.5–200	0.5

**Table 3:** Intra- and interday precision (RSD, %) and accuracy (bias, %) of QC samples.

Compound	Nominal concentration (ng/mL)	Intraday			Interday		
		Observed concentration (ng/mL)	RSD (%)	Bias (%)	Observed concentration (ng/mL)	RSD (%)	Bias (%)
Promethazine	0.5	0.52	12.57	4.56	0.50	16.97	1.01
	1.25	1.19	8.91	−4.02	1.17	11.22	−5.61
	12.5	11.79	6.56	−5.60	11.79	9.24	−5.61
	150	137.95	10.47	−8.02	140.96	13.31	−6.02

Promethazine sulfoxide	0.5	0.52	6.84	5.63	0.54	6.24	9.44
	1.25	1.26	1.37	1.23	1.28	6.10	2.56
	12.5	13.92	5.09	11.40	13.84	5.10	10.74
	150	138.65	3.54	−7.56	137.21	8.18	−8.52

Schisandrin	0.5	0.53	5.37	6.49	0.49	12.56	−1.95
	1.25	1.29	9.44	3.88	1.28	6.07	2.51
	12.5	13.09	6.15	4.75	13.17	6.25	5.43
	150	156.50	5.32	4.33	156.69	6.39	4.46

**Table 4:** Recovery and matrix effect of the three analytes in rat plasma.

Compound	Nominal concentration (ng/mL)	Recovery (%)			Matrix effect (%)		
		Recovery	SD	Bias (%)	Matrix	SD	Bias (%)
Promethazine	1.25	89.81	5.04	5.62	104.36	6.35	6.08
	12.5	87.17	2.44	2.80	103.74	3.24	3.13
	150	84.50	5.26	6.23	102.37	1.57	1.53

Promethazine sulfoxide	1.25	99.24	4.84	4.88	121.56	8.10	6.67
	12.5	96.70	4.74	4.90	122.80	16.34	13.31
	150	100.44	3.19	3.18	122.06	10.09	8.27

Schisandrin	1.25	97.92	5.07	5.17	105.19	11.70	11.12
	12.5	99.79	10.43	10.45	109.68	14.98	13.66
	150	98.65	4.31	4.37	106.51	10.63	9.98

**Table 5:** Stability of the analytes in rat plasma under different storage conditions.

Compound	Nominal concentration (ng/mL)	Short-term storage (4 h)		Autosampler stability (24 h)		Freeze-thaw stability	
		RSD (%)	Bias (%)	RSD (%)	Bias (%)	RSD (%)	Bias (%)
Promethazine	0.5	12.93	−1.68	4.90	4.96	10.87	−0.83
	1.25	12.91	−9.38	12.46	10.59	11.58	−3.21
	12.5	9.01	−1.44	14.55	6.20	8.20	−2.41
	150	6.37	6.10	9.39	−6.50	2.89	2.074

Promethazine sulfoxide	0.5	8.039	−4.51	7.74	−1.48	5.38	6.71
	1.25	7.87	−6.86	3.59	7.19	5.47	1.48
	12.5	6.52	−1.58	7.88	11.41	2.26	3.78
	150	7.71	−5.23	3.78	−10.28	2.63	−8.28

Schisandrin	0.5	13.14	−1.88	10.01	−13.03	17.20	−5.05
	1.25	11.09	−3.29	5.35	−6.60	4.40	−9.02
	12.5	2.74	−1.92	1.55	−6.62	4.36	−1.19
	150	1.84	−2.24	10.52	−11.38	3.92	−2.56

**Table 6:** Pharmacokinetic parameters of PMZ and PMZSO in rats after oral administration of PMZ with or without *S. chinensis* (*n* = 6-7).

Compound	Group	AUC (0-*t*) (ng h/mL)	*t*_1/2_ (h)	*T*_max_ (h)	*C*_max_ (ng/mL)
Promethazine	Promethazine	37.53 ± 9.54	5.92 ± 3.84	0.36 ± 0.13	14.35 ± 6.17
	PMZ + WWZ	42.72 ± 12.96	6.55 ± 3.16	6.11 ± 3.45^*∗*^	6.96 ± 2.12^*∗*^
	PMZ + WWZ for 3 weeks	24.76 ± 3.45^*∗*^	3.47 ± 0.97	5.67 ± 0.82^*∗*^	3.59 ± 0.57^*∗*^

Promethazine sulfoxide	Promethazine	179.33 ± 56.01	3.35 ± 1.33	3.43 ± 1.51	23.37 ± 5.33
	PMZ + WWZ	177.52 ± 30.35	2.55 ± 0.41^*∗*^	5.67 ± 1.51	25.58 ± 9.24
	PMZ + WWZ for 3 weeks	156.14 ± 26.04	2.72 ± 0.59	4.00 ± 1.15	23.86 ± 3.47

^*∗*^ *p* < 0.05 indicates significant differences from the PMZ group.

**Table 7:** Pharmacokinetic parameters of schisandrin in rats after oral administration of the *S. chinensis* water extract once or for continuously three weeks, coadministered with or without PMZ (*n* = 6-7).

Group	AUC (0-*t*) (ng h/mL)	*t*_1/2_ (h)	*T*_max_ (h)	*C*_max_ (ng/mL)
WWZ	248.60 ± 81.52	2.86 ± 1.39	6.00 ± 1.26	43.60 ± 16.24
PMZ + WWZ	60.74 ± 22.15^*∗*^	5.05 ± 1.75^*∗*^	4.79 ± 2.48	12.52 ± 10.54^*∗*^
WWZ for 3 weeks	69.95 ± 44.45^*∗*^	3.43 ± 1.96	3.50 ± 2.17^*∗*^	16.90 ± 14.41^*∗*^
PMZ + WWZ for 3 weeks	113.01 ± 56.70^*∗*^	3.44 ± 1.62	4.33 ± 1.97	29.70 ± 19.68

^*∗*^ *p* < 0.05 indicates significant differences from the WWZ group.

## Data Availability

The data used to support the findings of this study are available from the corresponding author upon request.

## Supplementary Materials

Supplementary Materials Supplementary Figure 1: the MRM spectrum of each component: (A) highest calibration samples (200 ng/mL) and (B) blank samples. 1: general spectrum; 2: schisandrin; 3: bifendate; 4: PMZSO; 5: PMZ; 6: metronidazole.
