# Supplementary material for: Simultaneous Determination of Schisandrin and Promethazine with Its Metabolite in Rat Plasma by HPLC-MS/MS and Its Application to a Pharmacokinetic Study
Source: Int J Anal Chem. 2019 Dec 9;2019:3497045. doi: 10.1155/2019/3497045 (PMC6925819; doi:10.1155/2019/3497045)
Supplement: Supplementary Materials — Supplementary Figure 1: the MRM spectrum of each component: (A) highest calibration samples (200 ng/mL) and (B) blank samples. 1: general spectrum; 2: schisandrin; 3: bifendate; 4: PMZSO; 5: PMZ; 6: metronidazole. [file 3497045.f1.docx]

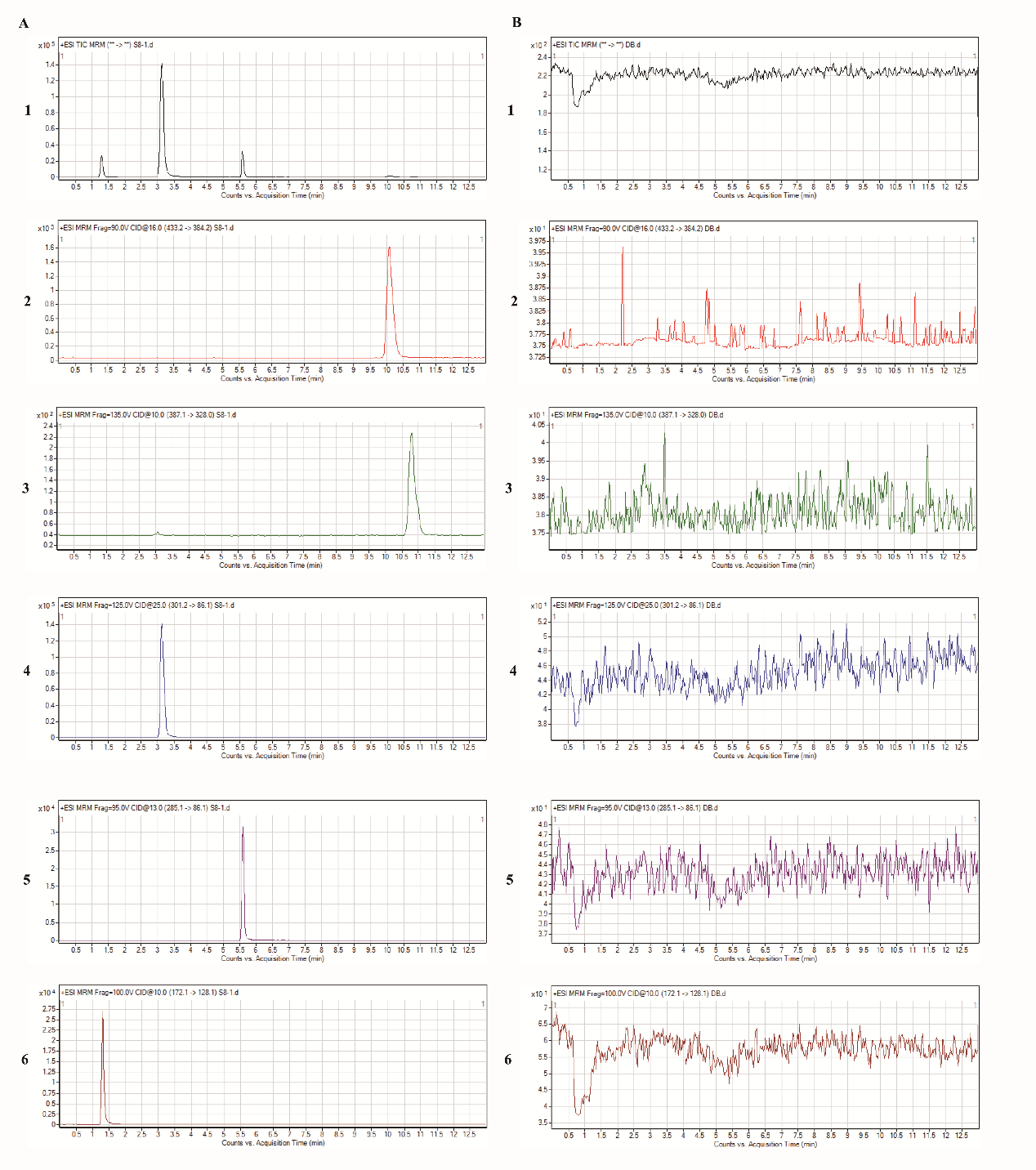


Supplementary Figure 1. The MRM spectrum of each component (A) highest calibration samples (200 ng/mL) and (B) blank samples. (1) General spectrum; (2) Schisandrin; (3) Bifendate; (4) PMZSO; (5) PMZ; (6) Metronidazole.
